# Supplementary material for: Content-rich biological network constructed by mining PubMed abstracts
Source: BMC Bioinformatics. 2004 Oct 8;5:147. doi: 10.1186/1471-2105-5-147 (PMC528731; doi:10.1186/1471-2105-5-147)
Supplement: Additional File 2 — The original results of the above study (non-essential files are deleted to keep the file size under the limit set by BMC bioinformatics). [file 1471-2105-5-147-S2.bz2 › chilibotAdditionalFile2/dip40/103ID7937747E433/html/MCL1_BCL2L1.html]

 


 **MCL1** and **BCL2L1** 
  
Found 182 abstracts in PubMed, retrieved 40.  
 

 What does Google say? 
 PDF only 
| .edu only 

---

**Interactive relationship** (e.g. stimulation, inhibition, etc)

**Neutral relationship**- the elimination of Mcl 1  [ **MCL1** ]  is required for the translocation of Bcl xL  [ **BCL2L1** ] .  Ref: 12783855 Genes Dev< MedlineTA>Genes Dev,
- gossypol induced apoptosis triggers down regulation of anti apoptosis Bcl 2 members Bcl X  [ **BCL2L1** ]  L, Bag 1 and Mcl 1  [ **MCL1** ] , up regulation of pro apoptosis Bcl 2 member Bak, activation of caspase,, and 9, up regulation of Apaf 1, release of cytochrome c cyto c from mitochondria, and activation of both DFF45 and PARP.  Ref: 12818369 Biochem Pharmacol, 2003

**Non-interactive relationship** (e.g. studied together, co-existance, homology, etc.)

- Using immunohistochemical methods and a 10% cut off, each protein was detected in a subset of PTCLs BCL 2 in 46%, BCL XS  [ **BCL2L1** ]  in 49%, BAX in 57%, BCL XL  [ **BCL2L1** ]  in 57%, and MCL 1  [ **MCL1** ]  in 65%.  Ref: 12754745 J Pathol, 2003
- **BCL2L1**  was underexpressed in CLL and CML, but not in AML, ALL and MCL  [ **MCL1** ] .  Ref: 12580957 Br J Haematol, 2003
- The latter include Bcl 2, Bcl X  [ **BCL2L1** ]  L, Mcl 1  [ **MCL1** ] , NF K B, survivin, livin, and ML IAP.  Ref: 12579529 J Pathol, 2003
- This study examined cutaneous T cell lymphoma CTCL cell lines and cutaneous lesions for the presence of Bcl 2 gene family members and found that the two apoptosis inhibiting members Bcl xL  [ **BCL2L1** ]  and Mcl 1  [ **MCL1** ]  and the two apoptosis supporting members Bad and Bax were expressed.  Ref: 12754746 J Pathol, 2003
- The levels of Bcl X  [ **BCL2L1** ]  L and Mcl 1  [ **MCL1** ]  remain unchanged.  Ref: 12847548 Genes Immun, 2003
- The study aim was to investigate Stat 3 activation in a series of multiple myeloma MM cases and its effect on downstream targets such as the anti apoptotic proteins Bcl xL  [ **BCL2L1** ] , Mcl 1  [ **MCL1** ] , and Bcl 2, and the cell cycle protein cyclin D1.  Ref: 12707028 Am J Pathol, 2003
- 2 Mcl 1  [ **MCL1** ]  and Bcl X  [ **BCL2L1** ]  L, which can override apoptosis.  Ref: 12579529 J Pathol, 2003
- Immunohistochemistry was performed on paraffin sections using antibodies against cyclin D1, Bcl 2, Bcl xL  [ **BCL2L1** ] , Mcl 1  [ **MCL1** ] , p21, Stat 3, and Stat 3 phosphorylated P.  Ref: 12707028 Am J Pathol, 2003
- this is paralleled by higher Bcl 2, Bcl xL  [ **BCL2L1** ] , Mcl 1  [ **MCL1** ] , Pgp and lower Bax expression levels.  Ref: 12745269 Haematologica, 2003
- These inhibitors were found to be Mcl 1  [ **MCL1** ]  and Bcl xL  [ **BCL2L1** ] , two antiapoptotic members of the Bcl 2 family.  Ref: 12783855 Genes Dev< MedlineTA>Genes Dev, 2003
- We therefore developed a four color flow cytometry method that enables establishment of apoptosis related protein expression such as Bcl 2, Bcl x  [ **BCL2L1** ]  L, Mcl 1  [ **MCL1** ]  and Bax at diagnosis and in MRD.  Ref: 12682637 Leukemia, 2003
- Western blot analysis was performed with anti ERK1 2, Mcl 1  [ **MCL1** ] , Bak, Bcl x  [ **BCL2L1** ]  and Bax antibodies.  Ref: 12606824 Mol Med, 2002
- Furthermore, the anti apoptotic proteins Bcl 2, Mcl 1  [ **MCL1** ]  and Bcl xL  [ **BCL2L1** ]  were down regulated, while the expression of the pro apoptotic Bax protein was increased.  Ref: 12366710 Eur J Haematol, 2002
- Janus kinase inhibitor AG490 inhibits Stat3 activation with a concomitant reduction in steady state levels of Bcl X  [ **BCL2L1** ]  L, Bcl 2 and Mcl 1  [ **MCL1** ]  proteins and induces apoptosis in U251 cells as revealed by Poly ADP ribose polymerase cleavage and Annexin V staining.  Ref: 12466961 Oncogene, 2002
- We report herein that human oocytes and preimplantation embryos possess abundant levels of transcripts encoding cell death suppressors, Mcl 1  [ **MCL1** ] , Bcl x  [ **BCL2L1** ]  and Bag 1, and the cell death inducer genes, Bax and Caspase 2.  Ref: 12606589 Mol Hum Reprod, 2003
- All cell lines express high levels of cyclin E, c Myc, Bcl 2, Bax, Bcl x  [ **BCL2L1** ]  L, and Mcl 1  [ **MCL1** ] .  Ref: 12683869 Arch Pathol Lab Med, 2003
- Our aim in this study is to investigate which anti apoptotic Bcl 2 family proteins Bcl 2, Bcl XL  [ **BCL2L1** ] , Mcl 1  [ **MCL1** ]  and which signal transduction pathway JAK STAT, Ras MAPK, PI 3K Akt can mediate the anti apoptotic effect of IL 6 on a human myeloma cell line XG 7.  Ref: 12479056 Ai Zheng, 2002
- The Bcl x  [ **BCL2L1** ] , Mcl 1  [ **MCL1** ] , Bad, and Bax proteins were also expressed in all CTCL skin lesions tested.  Ref: 12754746 J Pathol, 2003
- In contrast to Bcl xL  [ **BCL2L1** ]  and Mcl 1  [ **MCL1** ] , the expression of Bcl 2 showed an inverse correlation with proliferation rate P 0.0003.  Ref: 12707028 Am J Pathol, 2003
- Following UV treatment, Mcl 1  [ **MCL1** ]  protein synthesis is blocked, the existing pool of Mcl 1  [ **MCL1** ]  protein is rapidly degraded by the proteasome, and cytosolic Bcl xL  [ **BCL2L1** ]  translocates to the mitochondria.  Ref: 12783855 Genes Dev< MedlineTA>Genes Dev, 2003
- The activation state of protein kinase ERK, which is involved in Ras MAPK signal transduction pathway mediating cell survival and proliferation, and the expression of anti apoptotic Bcl 2 family proteins Bcl 2, Bcl x  [ **BCL2L1** ]  L and Mcl 1  [ **MCL1** ]  in Sko 007 cells with or without IFNalpha were determined by immunoblot assay.  Ref: 12482347 Zhonghua Xue Ye Xue Za Zhi, 2002
- Remarkably, these events occurred in the absence of any reduction in the expression of the Bcl 2 family members Bcl 2, Mcl 1  [ **MCL1** ]  and Bcl XL  [ **BCL2L1** ] , or any change in the proapoptotic molecules Bad or Bax.  Ref: 12842827 Am J Physiol Gastrointest Liver Physiol< MedlineTA>Am J Physiol Gastrointest Liver Physiol, 2002
- The proliferation index, assessed by the MIB 1 antibody, correlated with expression levels of MCL 1  [ **MCL1** ]  R = 0.42, p = 0.003, BCL 2 R = 0.32, p = 0.027, BAX R = 0.33, p = 0.014, and BCL XL  [ **BCL2L1** ]  R = 0.34, p = 0.015 Spearman rank.  Ref: 12754745 J Pathol, 2003
- The expression of Bcl 2, Bcl xL  [ **BCL2L1** ] , and Mcl 1  [ **MCL1** ]  may therefore be sufficient to guarantee the survival of malignant CTCL cells.  Ref: 12754746 J Pathol, 2003
- The present study investigated expression levels of the anti apoptotic proteins BCL 2, BCL XL  [ **BCL2L1** ]  and MCL 1  [ **MCL1** ]  and the pro apoptotic proteins BAX and BCL XS  [ **BCL2L1** ]  in a series of 112 peripheral T cell lymphomas PTCLs classified according to the WHO classification.  Ref: 12754745 J Pathol, 2003
- Treatment with FP also attenuated the mRNA and protein levels of XIAP, cIAP 2, Mcl 1  [ **MCL1** ] , Bcl x  [ **BCL2L1** ] , and survivin.  Ref: 12517783 Cancer Res, 2003
- Blockade of Src or Stat3 is also accompanied by down regulation of expression of the anti apoptotic genes, Bcl x  [ **BCL2L1** ]  L and Mcl 1  [ **MCL1** ] .  Ref: 12370822 Oncogene, 2002
- The expression of apoptosis related genes BCL2, BAX,  **BCL2L1** , BCL2A1,  **MCL1** , DAPK1 and MYC was studied by quantitative real time polymerase chain reaction on total RNA samples from patients with acute lymphoblastic leukaemia ALL, n = 16, acute myeloid leukaemia AML, n = 27, chronic myeloid leukaemia CML, n = 12, mantle cell lymphoma MCL  [ **MCL1** ] , n = 19 and chronic lymphoid leukaemia CLL, n = 32.  Ref: 12580957 Br J Haematol, 2003
- It was revealed that LIGHT treatment resulted in down regulation of anti apoptosis Bcl 2 family member Bcl 2, Bcl X  [ **BCL2L1** ]  L, Bag 1, and Mcl 1  [ **MCL1** ] .  Ref: 12767529 Cancer Lett, 2003
- Treatment with G3139 led to a sequence specific reduction of Bcl 2 protein levels within 4 days of exposure in 10 out of 11 clinical samples from patients with chemosensitive and multidrug resistant disease, without significant reduction of alpha Actin, Bax, Bcl XL  [ **BCL2L1** ] , or Mcl 1  [ **MCL1** ]  proteins.  Ref: 12529680 Leukemia, 2003
- The Bcl 2, Mcl 1  [ **MCL1** ] , Bcl xL  [ **BCL2L1** ]  S, Bcl w, Bax, Bak, and Bad were shown to be expressed in both malignant and non neoplastic, normal plasma cells.  Ref: 12366710 Eur J Haematol, 2002
- Melanocytic cells expressed relatively high levels of Bcl 2, Bcl X  [ **BCL2L1** ]  L, Mcl 1  [ **MCL1** ] , C IAP 1, C IAP 2, XIAP, Livin, and Apaf 1.  Ref: 12535197 J Invest Dermatol, 2003
- Levels of the CREB dependent anti apoptotic protein Mcl 1  [ **MCL1** ]  and of the NF kappaB dependent anti apoptotic mediator Bcl x  [ **BCL2L1** ]  were significantly decreased in PI3 Kgamma neutrophils.  Ref: 12464394 Cell Signal, 2003
- Treatment with Epo B followed by FP induced more Bax conformational change and was associated with a greater decline in the levels of XIAP, cIAP 2, Mcl 1  [ **MCL1** ] , and Bcl x  [ **BCL2L1** ] .  Ref: 12517783 Cancer Res, 2003
- Although STAT3 activation is known to induce expression of Bcl 2 family proteins, PEL cell apoptosis was independent of Bcl 2, Bcl X  [ **BCL2L1** ]  L, or Mcl 1  [ **MCL1** ]  protein expression.  Ref: 12393476 Blood, 2003
- This increase in viability occurs independently of Bcl xL  [ **BCL2L1** ]  and Mcl 1  [ **MCL1** ]  expression though, two survival genes reported to be downstream of IL 6 signaling.  Ref: 12426000 Cell Immunol< MedlineTA>Cell Immunol, 2003
- High levels of anti apoptotic proteins Bcl xL  [ **BCL2L1** ]  and Mcl 1  [ **MCL1** ]  were identified in 89% and 100% of all cases, respectively.  Ref: 12707028 Am J Pathol, 2003
- Hypoxia induced cell cycle arrest at the G0 G1 phases and massive cell apoptosis after 24 hours through a reduction in the Bcl 2 to Bax ratio, downregulation of Bcl XL  [ **BCL2L1** ]  and Mcl 1  [ **MCL1** ] , and upregulation of caspase 3 and caspase 8.  Ref: 12746487 J Cell Sci< MedlineTA>J Cell Sci, 2003
- IL 6 rescues the hyporesponsiveness of c Rel deficient B cells independent of Bcl xL  [ **BCL2L1** ] , Mcl 1  [ **MCL1** ] , and Bcl 2.  Ref: 12426000 Cell Immunol< MedlineTA>Cell Immunol, 2003
- MM cells express multiple Bcl 2 family members, including Bcl 2, Bcl XL  [ **BCL2L1** ] , and Mcl 1  [ **MCL1** ] , which are thought to play a key role in the survival and drug resistance of myeloma.  Ref: 12429644 Clin Cancer Res, 2002
- Immunohistochemistry was used on paraffin sections to detect LMP 1 EBV, CD15 and the apoptosis related proteins bcl 2, bax, bcl X  [ **BCL2L1** ] , mcl 1  [ **MCL1** ]  and CD95.  Ref: 12688319 Leuk Lymphoma, 2003
- RNA and protein analyses revealed several candidate target genes of Stat5, including Bcl x  [ **BCL2L1** ] , Mcl 1  [ **MCL1** ] , c Myc and cyclin D2, which were down regulated after treatment with PD180970.  Ref: 12483533 Oncogene, 2002
- VD3 induced decreases in levels of antiapoptotic proteins Bcl 2, Bcl X  [ **BCL2L1** ] , and Mcl 1  [ **MCL1** ] , BAG1L, XIAP, cIAP1, and cIAP2 in association with increases in apoptosis.  Ref: 12479363 Mol Cancer Ther, 2002
- BCL2,  **BCL2L1** , BCL2A1 and  **MCL1**  were overexpressed and DAPK1 was underexpressed in CLL samples with a 11q23 deletion.  Ref: 12580957 Br J Haematol, 2003
- Taken together, these findings suggest that the superior sequence dependent anti breast cancer activity of Epo B followed by FP may be due to FP induced Bax conformational change and down regulation of the antiapoptotic IAP, Bcl x  [ **BCL2L1** ] , and Mcl 1  [ **MCL1** ]  proteins, but this treatment may not overcome the resistance to apoptosis of breast cancer cells conferred by overexpression of Bcl 2.  Ref: 12517783 Cancer Res, 2003
- All multidomain anti apoptotic members Bcl x  [ **BCL2L1** ] , Bcl 2, Mcl 1  [ **MCL1** ] , Bcl w and Bcl2l10 Diva Boo except Bcl2a1 A1 are expressed in OLC.  Ref: 12787069 J Neurochem, 2003
- ... the FP mediated decline in the levels of IAP proteins, Mcl  [ **MCL1** ]  11 and Bcl x  [ **BCL2L1** ]  remained unaltered.  Ref: 12517783 Cancer Res, 2003
- Semiquantitative and real time RT PCR revealed that Bcl xL  [ **BCL2L1** ]  and Mcl 1  [ **MCL1** ]  mRNAs are the dominant anti apoptotic members and increase four and twofold, respectively, with maturation.  Ref: 12787069 J Neurochem, 2003
- This increased apoptosis was accompanied by decreased antiapoptotic bcl 2 mRNA expression among bcl 2 related genes bcl 2, bax, bak, mcl 1  [ **MCL1** ] , and bcl X  [ **BCL2L1** ]  L S, and the effect was also more prominent in the cagA strains.  Ref: 12540563 Infect Immun, 2003
